# Causal Evidence for the Multiple Demand Network in Change Detection: Auditory Mismatch Magnetoencephalography across Focal Neurodegenerative Diseases

**DOI:** 10.1523/JNEUROSCI.1622-21.2022

**Published:** 2022-04-13

**Authors:** Thomas E. Cope, Laura E. Hughes, Holly N. Phillips, Natalie E. Adams, Amirhossein Jafarian, David Nesbitt, Moataz Assem, Alexandra Woolgar, John Duncan, James B. Rowe

**Affiliations:** ^1^Department of Clinical Neurosciences, University of Cambridge, Cambridge CB2 0SZ, United Kingdom; ^2^Cognition and Brain Sciences Unit, Medical Research Council, Cambridge CB2 7EF, United Kingdom; ^3^Cambridge Centre for Ageing and Neuroscience (Cam-CAN), University of Cambridge, Cambridge CB2 7EF, United Kingdom; ^4^Department of Experimental Psychology, University of Oxford, Oxford OX1 3UD, United Kingdom; ^5^Cambridge University Hospitals NHS Trust, Cambridge CB2 0SZ, United Kingdom

**Keywords:** Alzheimer's disease, bvFTD, dementia, dynamic causal modeling, mismatch negativity, multiple demand

## Abstract

The multiple demand (MD) system is a network of fronto-parietal brain regions active during the organization and control of diverse cognitive operations. It has been argued that this activation may be a nonspecific signal of task difficulty. However, here we provide convergent evidence for a causal role for the MD network in the “simple task” of automatic auditory change detection, through the impairment of top-down control mechanisms. We employ independent structure-function mapping, dynamic causal modeling (DCM), and frequency-resolved functional connectivity analyses of MRI and magnetoencephalography (MEG) from 75 mixed-sex human patients across four neurodegenerative syndromes [behavioral variant fronto-temporal dementia (bvFTD), nonfluent variant primary progressive aphasia (nfvPPA), posterior cortical atrophy (PCA), and Alzheimer's disease mild cognitive impairment with positive amyloid imaging (ADMCI)] and 48 age-matched controls. We show that atrophy of any MD node is sufficient to impair auditory neurophysiological response to change in frequency, location, intensity, continuity, or duration. There was no similar association with atrophy of the cingulo-opercular, salience or language networks, or with global atrophy. MD regions displayed increased functional but decreased effective connectivity as a function of neurodegeneration, suggesting partially effective compensation. Overall, we show that damage to any of the nodes of the MD network is sufficient to impair top-down control of sensation, providing a common mechanism for impaired change detection across dementia syndromes.

**SIGNIFICANCE STATEMENT** Previous evidence for fronto-parietal networks controlling perception is largely associative and may be confounded by task difficulty. Here, we use a preattentive measure of automatic auditory change detection [mismatch negativity (MMN) magnetoencephalography (MEG)] to show that neurodegeneration in any frontal or parietal multiple demand (MD) node impairs primary auditory cortex (A1) neurophysiological response to change through top-down mechanisms. This explains why the impaired ability to respond to change is a core feature across dementias, and other conditions driven by brain network dysfunction, such as schizophrenia. It validates theoretical frameworks in which neurodegenerating networks upregulate connectivity as partially effective compensation. The significance extends beyond network science and dementia, in its construct validation of dynamic causal modeling (DCM), and human confirmation of frequency-resolved analyses of animal neurodegeneration models.

## Introduction

The multiple demand (MD) system is a network of brain regions engaged in flexible organization and control of cognitive operations across diverse mental activities (Duncan, 2010; Duncan et al., 2020). MD robustly comprises domain general regions in middle frontal gyrus, inferior frontal sulcus, anterior insula, and intraparietal sulcus, as well as supplementary motor area and anterior cingulate cortex (Fedorenko et al., 2013). Within the complex taxonomy of functional brain networks (Uddin et al., 2019), this lateral and ventral fronto-parietal network has been variously named for its role in control (Dosenbach et al., 2008; Vincent et al., 2008) and executive function (Seeley et al., 2007; Niendam et al., 2012), is associated with both fluid intelligence and spatial working memory (Assem et al., 2020b), and has been implicated in executive abnormalities in logopenic Alzheimer's disease (Ramanan et al., 2020). MD encompasses nodes that are modulated by alterations in behaviorally relevant stimuli (Desimone and Duncan, 1995; Corbetta and Shulman, 2002; Macaluso and Driver, 2005), and overlaps with frontal and parietal regions sometimes included in the ventral attention network (Corbetta et al., 2008; Macaluso, 2010), but is distinct from the cingulo-opercular and salience networks (Power et al., 2011; Assem et al., 2020a; Dworetsky et al., 2021). MD can be activated by simple tasks (Crittenden and Duncan, 2014), but increases in activity with task complexity (Wen et al., 2018) and the difficulty of stimulus discrimination (Woolgar et al., 2011). It is especially engaged in tasks with high attentional demand (Jung et al., 2021), but it is also visible in resting state functional connectivity patterns (Smith et al., 2009; Power et al., 2011; Gordon et al., 2017; Dworetsky et al., 2021). While the activation of this domain-general cognitive core is well established and precisely delineated (Assem et al., 2020a) some argue that this is a nonspecific signal of task difficulty, rather than playing a direct perceptual processing role (Diachek et al., 2020).

Here, we go beyond this associative evidence, and test whether the MD network plays a causal role in automatic sensory change detection, when there is no required response, explicit decision, or attentional demand, through top-down hierarchical influences on auditory brain regions. To do this, we recorded magnetoencephalography (MEG) during an auditory mismatch [mismatch negativity (MMN)] paradigm from 75 patients across four neurodegenerative syndromes, and 48 age-matched controls. We provide causal evidence by examining the differential effects of chronic network perturbations because of neurodegeneration in separable network nodes (Wolff and Ölveczky, 2018). The graded nature of neurodegenerative disease allows us to do this parametrically, providing information about the function of impaired nodes that would be impossible to observe if they were completely lesioned, for example from stroke. We first demonstrate with structure-function mapping that damage to MD nodes, but not other core cognitive networks, reduces the neurophysiological response to auditory change. We then characterize the nature of the effective connectivity disruption with dynamic causal modeling (DCM) of the evoked response, which reveals the consequences of damage, and the partially effective compensation from other nodes. Finally, we demonstrate with independent functional connectivity analyses of the induced response that the frequency specificity of compensatory upregulation is in-keeping with animal models and previous human evidence, providing concurrent criterion and construct validity for the DCM. Together, these analyses demonstrate causality by showing that frontal, parietal, and auditory regions are functionally and effectively connected during automatic sensory change detection, and that damage to frontal or parietal regions specifically reduces the neurophysiological response to change by altering network dynamics.

## Materials and Methods

### Participants

Seventy-five patients with neurodegenerative syndromes were recruited according to consensus clinical criteria (Table 1). These comprised 23 patients with behavioral variant fronto-temporal dementia (bvFTD; Rascovsky et al., 2011), 10 patients with nonfluent variant primary progressive aphasia (nfvPPA; Gorno-Tempini et al., 2011), and 15 patients with posterior cortical atrophy (PCA; Crutch et al., 2017). Each of these patients with focal degeneration was individually matched to a healthy control of the same age and gender, from the volunteer panel of the MRC Cognition and Brain Sciences Unit, or the population-based Cambridge Center for Ageing and Neuroscience study (Shafto et al., 2014). A fourth group with early Alzheimer's Disease (AD) pathology was recruited to assess the general applicability and specificity of our results in the context of more diffuse pathology. Twelve patients with AD (McKhann et al., 2011) and 15 with amnestic mild cognitive impairment (MCI) and a positive amyloid PET scan (Klunk et al., 2004; Dubois et al., 2016) were included. Seven patients with amnestic MCI were recruited but had negative amyloid PET scans and were not analyzed.

**Table 1.** Participant demographics

	All focal patients	Healthy controls	bvFTD	nfvPPA	PCA	ADMCI
*N*	48	48	23	10	15	27
Age	63.6 ± 7.99	63.6 ± 8.19	61.8 ± 5.45	71.6 ± 9.11	62.5 ± 8.43	73.4 ± 8.68
Gender	26 F 22 M	26 F 22 M	13 F 12 M	7 F 3 M	9 F 7 M	14 F 13 M
MMSE	23.0 ± 1.8	29.3 ± 0.85	23.2 ± 6.3	27.8 ± 2.3	19.1 ± 5.7	24.4 ± 3.4
ACE-R	66.3 ± 6.2	95.9 ± 3.4	67.0 ± 19.5	84.5 ± 11.5	54.5 ± 23.2	73.2 ± 11.9

bvFTD, behavioral variant fronto-temporal dementia; nfvPPA, nonfluent variant primary progressive aphasia; PCA, posterior cortical atrophy; ADMCI, Alzheimer's disease mild cognitive impairment with positive amyloid imaging.

All participants underwent neuropsychological assessment with the revised Addenbrooke's cognitive examination (ACE-r; Mioshi et al., 2006) and the mini mental state examination (MMSE). They had a standardized T1 structural MRI scan at 3T within two months of their MEG session.

Participants gave written informed consent, and ethical approval for the study was given by Suffolk Research Ethics Committee (REC reference 07/H0307/64). Ethical approval for the Cam-CAN study was obtained from the Cambridgeshire 2 Research Ethics Committee (REC reference 10/H0308/50).

### Structural MRI voxel-based morphometry analysis

Data analysis scripts for this section are available at https://github.com/thomascope/MMN/blob/master/ICA_denoise/VBM/Master_Script_VBM.m and used a statistical parametric mapping approach in SPM12 r6906 (SPM, Wellcome Trust Center for Neuroimaging, University College London).

Subjects' individual anatomic T1-weighted MRI images (3D MPRAGE sequence, TR = 2300 ms, TE = 2.86 ms, inversion time 900 ms, flip angle 9°, field-of-view 192 × 192 × 144, 1.250mm slice thickness, collected on a 3T Siemens Tim Trio scanner) were first aligned by coregistration to an average image in MNI space, before segmentation and calculation of total intracranial volume (TIV). After segmentation, a study-specific DARTEL template was created from all study scans using default parameters. The templates were affine aligned to the SPM standard space using “Normalize to MNI space” and the transformation applied to individual modulated gray-matter segments, with an 8-mm FWHM Gaussian smoothing kernel.

The resulting images were entered into a general linear model with a single regressor for each of the five groups, and age and TIV as covariates of no interest, with an explicit gray-matter mask constructed from the control participants with 80% group agreement at a probability threshold of 5%. This model was estimated in two ways, to quantify both atrophy and Bayesian evidence for no atrophy in each group (i.e., to highlight normal as well as abnormal cortex). First, a classical estimation was performed to assess for group difference and thresholded for visualization as “hot” colors in Figure 2, both voxelwise multiple-comparison corrected with restricted maximum likelihood (REML) at FWE *p* < 0.05, and uncorrected at *p* < 0.001. Second, a Bayesian estimation was performed on the same model, and a Bayesian contrast between each patient group and controls specified. The resulting Bayesian map was subjected to hypothesis testing for the null in SPM12, resulting in a map of the posterior probability of the null at each voxel (indicating evidence of no atrophy). This map was thresholded for posterior probabilities for the null above 0.7 and cluster volumes of >1 cm^3^, consistent with our previous practice (Cope et al., 2017), for visualization in as “cool” colors in the right hand column of Figure 2.

### Auditory oddball paradigm

We exposed participants to a stereotyped auditory stimulus train containing “oddballs” that differed from standard tones in the location, intensity, duration, frequency or continuity domains (Näätänen et al., 2004), while they watched a silent movie. We used the “Optimum-1” type multi-MMN paradigm (Näätänen et al., 2004; Hughes et al., 2013) to investigate MMN responses to multiple deviant types. In this time-efficient variant of the classic oddball paradigm, standard tones are alternated with tones that deviate in one of five dimensions, while holding the other four stimulus properties constant. This paradigm has the advantage compared with traditional MMN paradigms that an equal number of standard and deviant evoked responses are obtained. Deviant types were presented pseudo-randomly, such that they never repeated consecutively and each deviant type appears at least once in a sequence of 10 tones. In this way, the occurrence of a deviant tone was entirely predictable, but the way in which it differed from the standard was entirely unpredictable. Duration deviants shortened the standard tone from 75 ms (including 7-ms cosine up and down ramps) to 25 ms. Intensity deviants altered the standard tone ±6 dB from 60 dB HL. Frequency deviants moved the standard tone, which comprised three harmonics of 500, 1000, and 1500 Hz, up or down by 10% to harmonics with fundamentals of 450 or 550 Hz. Laterality deviants presented the standard tone monaurally left or right, rather than binaurally. Finally, gap deviants had a 25-ms silent interval in the middle of a standard tone.

Tones were presented every 500 ms in three blocks of 5 min while participants watched a silent movie (BBC Planet Earth series 1, episode 9: Shallow Seas). There was no task. Each block began with fifteen standard tones, which were not included in later analyses. In total, 900 standard and 900 deviant tones (180 of each type) were presented using E-Prime 2.0 (Psychology Software Tools) via plastic tubes and earpieces. Participant hearing level was checked immediately before paradigm delivery using the same equipment and an automated two-down, one-up procedure to ensure that the stimuli were comfortable and audible.

### MEG acquisition and preprocessing

MEG recordings were completed at the MRC Cognition and Brain Sciences Unit using the Elekta Neuromag Vectorview System (Elekta Neuromag). The Vectorview system contained 102 sensor locations, each with one magnetometer and two orthogonal gradiometer superconducting thin-film sensors. Participant head movement was recorded during the MEG recording using five Head-Position Indicator (HPI) coils. Vertical and horizontal eye movements were recorded using two pairs of electrooculogram (EOG) electrodes. We used a 3D digitizer (Fastrak Polhemus) to digitize the HPI coils and >100 scalp points, all in relation to the nasion and bilateral preauricular anatomic fiducial points.

Data analysis scripts for all MEG analyses are available at https://github.com/thomascope/MMN/blob/master/ICA_denoise/Integrated_Pipeline.m.

Signal-space separation (Taulu et al., 2005) in Neuromag Maxfilter 2.2 was used to realign raw MEG data, separated from environmental noise, and compensated for head movement based on the tracked 3D position of the five HPI coils. Data were downsampled at this stage from 1 kHz to 250 Hz because of the extremely large size of our cohort and limits on available fileserver storage. Next, eye movements and blinks were automatically identified and rejected with separate independent component analyses for magnetometers and gradiometers performed in EEGlab (Swartz Center for Computational Neuroscience, University of California San Diego). Components were rejected if they were both significantly temporally correlated with contemporaneous electrooculography data and spatially correlated with separately acquired template data for blinks and eye movements. The data were then preprocessed in SPM12 r6906 with the following steps: trials were defined and the data epoched −100–500 ms around the onset of the tone with baseline correction by subtraction of the mean of the −100- to 0-ms period; data were low-pass filtered below 100 Hz then notch filtered between 48 and 52 Hz to exclude electrical noise at 50 Hz using bidirectional Butterworth filters to obtain zero-phase distortion; blocks were merged, and trials were subjected to robust averaging (Wager et al., 2005) followed by repeat lowpass filtering at 100 Hz to remove the high-frequency noise that is introduced by robust averaging.

### Source reconstruction

Two identical source reconstructions were performed in SPM12. One on the time-domain data before averaging for frequency-domain connectivity analyses (Granger Causality, Imaginary Coherence, and Phase Locking Value), and another on the trial-averaged data for source-space statistical analyses and DCM.

The forward model (lead field) was estimated from a single shell cortical mesh of each participant's individual anatomic T1-weighted MRI image. The cortical mesh was coregistered to the MEG data using the digitized fiducial and scalp points. We computed the inverse source reconstruction across the whole brain using the sLORETA algorithm (Pascual-Marqui, 2002; Jatoi et al., 2014) and extracted the local field potentials (LFPs) from 5-mm radius spheres in literature-defined regions of interest (ROIs) previously identified in MMN connectivity (Garrido et al., 2009a; Dietz et al., 2014): bilateral primary auditory cortex (A1; MNI coordinates: [−42 −22 7], [46 −14 8]), superior temporal gyrus (STG; [−61 −32 8], [59 −25 8]), inferior frontal gyrus (IFG; [46 20 8], [−46 20 8]), and inferior parietal cortex (IPC; [−49 −38 38], [57 −38 42]. We used literature defined co-ordinates to avoid double-dipping our dataset, but all IFG and IPC nodes and no A1 or STG nodes fell within probabilistic maps of the MD network (Dworetsky et al., 2021). The LFP data were then baseline corrected before statistical analysis.

### Evoked response analysis

For consistency with the previous literature (Hughes et al., 2013), we quantified MMN amplitude as the average mismatch response in left A1 between 100 and 200 ms. A control analysis comparing the amplitude of the M100 response to the standard tone, as defined by the magnitude of the peak deflection between 50 and 150 ms, was also performed to demonstrate that reductions in MMN amplitude were not simply because of reduced global signal in the patient populations.

### Structure-function relationship

To test the hypothesis that the MD network has a causal role in auditory change detection, structure-function maps were generated, and then compared with published MD functional parcellation maps. A regression general linear model was constructed in SPM to assess the relationship between gray matter volume and MMN amplitude. All participant scans were assessed as a single group against MMN amplitude, with age and TIV as covariates of no interest. To create the illustrative regression plots in Figure 7, filtered gray matter volume data were extracted from the SPM general linear model at voxels of peak effect, and plotted against MMN amplitude. Note that these locations were selected as they were already demonstrated to be significant statistical peaks, so these plots and the associated *r*-values merely illustrate effect size, without re-estimation of associated *p*-values.

Overlaps of cluster peak locations with binary maps from Shain et al. (2020) were assessed in MNI space with MRIcron. The neuromorphometrics atlas implemented within SPM was used to define anatomic ROIs.

Voxelwise overlap with probabilistic maps from Woolgar et al. (2018) and Dworetsky et al. (2021) were assessed in SPM (code at https://github.com/thomascope/MMN/blob/master/ICA_denoise/VBM/visualization_2020/propvox.m). First, the probabilistic maps were resliced into the same space as the structure-function correlation maps, then thresholded at 5%. These binary images were multiplied in imcalc by binarized structure-function correlation maps thresholded at *p* < 0.001 uncorrected. The proportion of surviving voxels from the thresholded structure-function was then calculated.

The normalized activation ratio map was calculated (code at https://github.com/thomascope/MMN/blob/master/ICA_denoise/VBM/visualization_2020/weightedvox.m) from the same resliced probabilistic maps, unthresholded, multiplied in imcalc with *t*-score structure-function correlation maps thresholded at *p* < 0.001 uncorrected. The total sum of all voxel values in this map was normalized by multiplication with the number of voxels in the gray matter mask used to create the structure-function correlation maps, followed by division by the product of the total sum of all voxel values in the probabilistic and thresholded structure-function maps. In this way, an activation ratio of between 0 and 1 was created, normalized against map size and range:

$$Normalizedactivationratio=\frac{(voxelt\_score,*voxelnetwork\_probability)*mask\_size}{\sum t\_score*\sum network\_probability}.$$

To ensure that our findings were not nonspecific group effects, we assessed the specificity of our results in several ways. In our original model, we checked that the structure-function analysis was not primarily implicating the most atrophic regions, nor was it implicating all the locations found to be atrophic. The Alzheimer's disease mild cognitive impairment with positive amyloid imaging (ADMCI) group, with predominant hippocampal/entorhinal atrophy, and the PCA group, with significant occipital lobe atrophy, are of particular interest here, as we do not implicate these regions as contributing to auditory change detection. Next, we ran a series of alternate regression models, each less sensitive but more specific in their assessment of differential neurodegeneration. First, we re-ran the analysis excluding the control participants. Although this reduces the between-subject variance in neurophysiological response, it does not assume that all differences are driven by the same loci. This is important, because the drivers of impaired change detection may be different for example in a patient with left frontal atrophy from nfvPPA and a patient with parieto-occipital atrophy from PCA. Indeed, if the MD network is working in an integrated fashion to support auditory change detection, atrophy in any of these areas should be sufficient to alter response. Second, we assessed a linear mixed model, with a factor “group” and two levels (controls, patients), with age and TIV as additional covariates of no interest. This answers the same question in a different way, assuming that the small amount of variance within the control group adds information and not noise. Finally, we assessed this linear mixed model with five “group” levels (controls, bvFTD, nfvPPA, PCA, ADMCI). This is the most specific analysis, but also potentially excludes effects of interest in areas of atrophy that are shared between patient groups. This is problematic for areas such as left superior parietal cortex, where no patient group had Bayesian evidence for no atrophy.

### DCM

We used DCM of evoked responses to identify the effective connectivity among eight neural sources of the MMN, and to assess the top-down influence of frontal and parietal brain regions on primary and association auditory regions. The models were fitted to the observed physiological responses, including responses to all tones and their modulation by deviance. We used biophysically informed canonical microcircuits (*spm_fx_cmc*) at each source to model the extrinsic connections between sources, and layer-specific intrinsic connections within each source among populations of pyramidal cells, spiny stellate cells and inhibitory interneurons (Moran et al., 2013). The canonical microcircuit model enables inferences about the mechanisms that generate the observed LFPs and their modulation by experimental stimuli (David et al., 2006; Kiebel et al., 2006, 2008; Muthukumaraswamy et al., 2015; Shaw et al., 2017).

**Figure 1.**
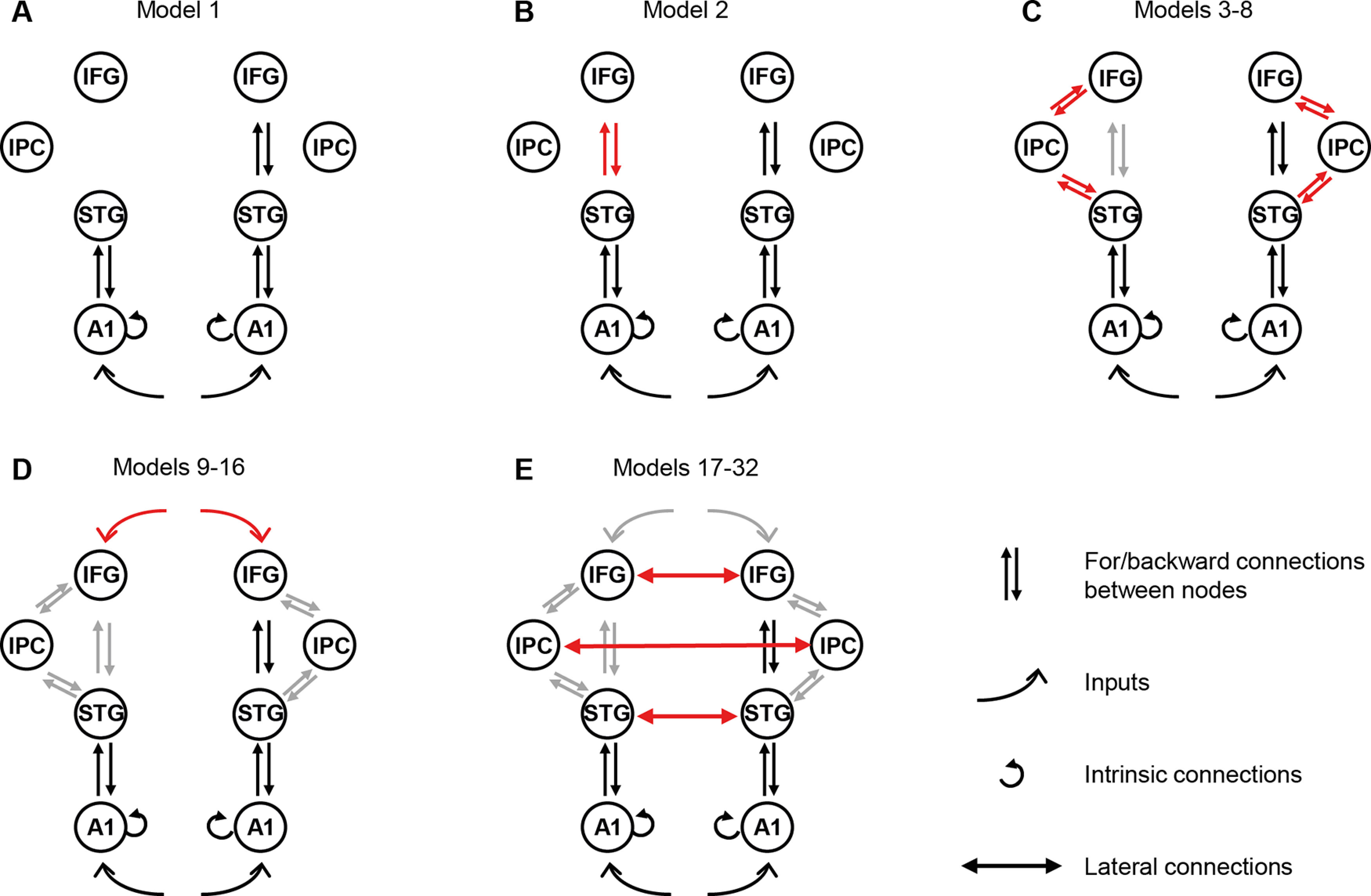
DCM model space. Nodes of interest were literature-specified locations in primary auditory cortex (A1), superior temporal gyrus (STG), inferior frontal gyrus (IFG), and inferior parietal cortex (IPC). Red arrows graphically indicate what has been added to each model compared with the previous, simpler model. ***A***, Model 1 is the original winning model from Garrido et al. (2009a). Subsequent models build from this with our alternative hypotheses based on the subsequent literature, to test our specific hypotheses. ***B***, First, we included connections between left STG and IFG (model 2). ***C***, Second, we alternated connections between left or bilateral IPC with STG and/or IFG (models 3–8). ***D***, Third, we added top-down IFG inputs to all previous models (models 9–16). ***E***, Finally, we repeated all the models with added lateral connections between STGs, IFGs, and IPCs (models 17–32).

We tested alternative hypotheses of the mechanism underlying the MMN through 32 generative models (Fig. 1). The model space was based on a combination of previous studies of the MMN (Giard et al., 1990; Doeller et al., 2003; Molholm et al., 2005; Garrido et al., 2009b; Dietz et al., 2014; Phillips et al., 2015). These models described the effective connectivity between temporal, frontal and parietal sources for the time window of 0–400 ms from the onset of each stimulus, encompassing the MMN interval. Input data were trial-averaged and Hanning windowed, with inversion frequency bounds of 4–48 Hz. All connections between MMN sources were bidirectional and modulated (Garrido et al., 2007; Phillips et al., 2016).

We built the model space from alternative hypothesis of network connections, building on the Garrido et al. (2009a) winning model (Fig. 1*A*) with driving inputs into bilateral A1 (MNI coordinates: [−42 −22 7], [46 −14 8]), with intrinsic connections within these sources, bidirectional connections between bilateral A1 and superior temporal gyri (STG, [−61 −32 8], [59 −25 8]), and bidirectional connections between right STG and right IFG ([46 20 8]). The second model (Fig. 1*B*) incorporated left IFG ([−46 20 8]), which has previously demonstrated greater evidence than model 1 for all deviant types in the Optimum-1 MMN paradigm in young healthy adults (Phillips et al., 2015). Models 3–8 (Fig. 1*C*) build on models 1–2 by adding bidirectional connections between STG and IPC ([−49 −38 38], [57 −38 42]; Dietz et al., 2014, models 3–4), between IPC and IFG (models 5–6), and between IPC and both IFG and STG (models 7–8). We repeated models 1–8 with inclusion of top-down temporal expectation inputs acting on the frontal source (Fig. 1*D*, models 9–16; Phillips et al., 2015, 2016; Chennu et al., 2016). Finally, models 17–32 (Fig. 1*E*) repeat models 1–16 with lateral connections between bilateral STGs, IFGs, and IPCs (Hughes et al., 2013).

### Model comparison

We used Bayesian model selection to compare the generative models (Penny et al., 2004) and to determine which model best explained the neural responses for each group (Kiebel et al., 2008; Stephan et al., 2009). We used random effects analysis to accommodate group heterogeneity (Stephan et al., 2010) with adjustment of the model fit for model complexity to reduce over-fitting (Kiebel et al., 2008). The free energy estimates of –log(model_evidence) were used to compare models and estimate the model exceedance probability (xp; that a single model was more likely to have generated the data compared with all other models). We report all models with an xp of >0.05.

Next, we compared eight model families using random effects Bayesian model averaging (Penny et al., 2010). Each model family contained four models differing in the presence or absence of top-down inputs to the system and cross-hemispheric connections, but with the same pattern of connectivity between STG, IFG, and IPC nodes. Specifically, referring to Figure 1, the fully connected family included models 8, 16, 24, and 32, the fully disconnected family models 1, 9, 17, and 25, with the other families being integer increments in between.

### Parametric empirical Bayes (PEB) analysis

We then compared the strength and modulation of DCM connectivity between controls and each patient group for the model with the highest probability using second level PEB analyses (*spm_dcm_peb*; Friston et al., 2015). This was performed twice; once for the “A-matrix,” or main effect of connectivity strength; and again for the “B-matrix,” the modulation strength or interaction between group and tone identity. These second level models were then subjected to Bayesian model comparison averaging with Bayesian model reduction (*spm_dcm_peb_bmc*). Differences in connection strength are displayed where Bayesian probability >0.95.

### Frequency resolved connectivity analyses

We extracted time series data from all sources, epoched between 0 and 500 ms after each tone. We then subtracted the condition-averaged waveform (i.e., the evoked response) in each source from every trial, resulting in data with zero-mean and approximate stationarity. We then calculated the Fourier spectra in FieldTrip using multitapers with a ±4-Hz smoothing box and subjected this decomposition to separate frequency-resolved connectivity analyses between every pair of connections in the winning DCM model, with partial imaginary coherence and partial phase-locking value. These measures were chosen as they are robust to source spread, and allow for each extrinsic connection to be individually examined, with the effect of all other sources partialled out. They are also robust to differences in signal-to-noise ratio between sources and individuals, but to further control for any effect of this the analyses were all repeated with the trial identities shuffled to create a null distribution, 100 times for every connection pair in every individual. Connectivity strength was averaged in the θ (4–8 Hz), α (8–20 Hz), β (20–30 Hz), low-γ (30–45 Hz), and high-γ (55–70 Hz) bands for between-group statistical comparisons.

## Results

### Patient groups show differential and graded patterns of atrophy

**Figure 2.**
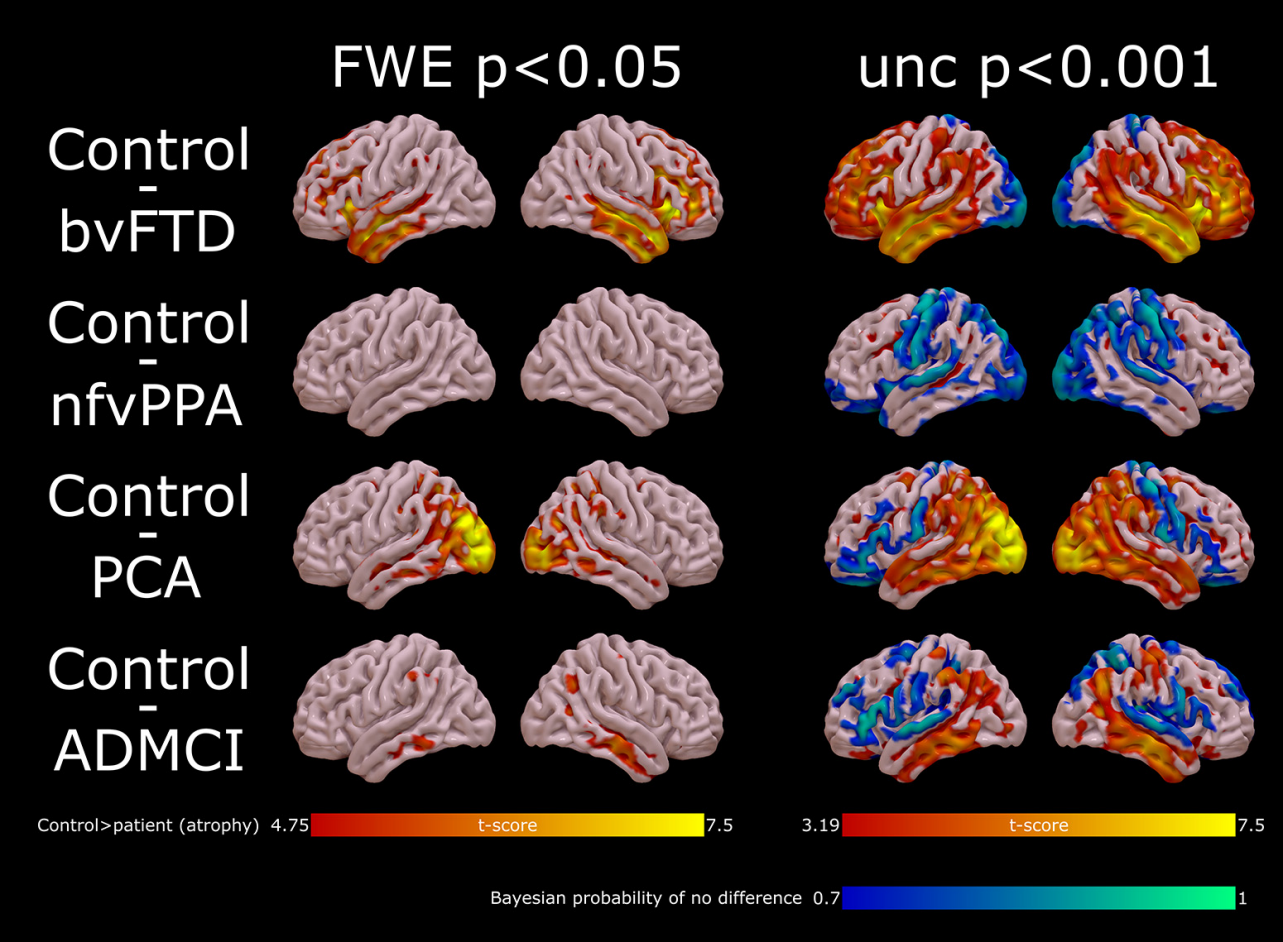
Distribution of gray matter atrophy in the patient participants. Random field theory t-maps for atrophy assessed with a classical statistical parametric mapping approach is shown in “hot” colors, REML thresholded at voxelwise FWE *p* < 0.05 (left) and uncorrected *p* < 0.001 (right). On the right-hand maps, Bayesian maps for posterior probability of no atrophy (the “null”) are also shown in “cool” colors, thresholded for illustration at 0.7. bvFTD, behavioral variant fronto-temporal dementia; nfvPPA, nonfluent variant primary progressive aphasia; PCA, posterior cortical atrophy; ADMCI, Alzheimer's disease mild cognitive impairment with positive amyloid imaging.

All patient groups showed atrophy distributions in keeping with expectations from previous literature (Fig. 2).

Patients with bvFTD showed marked atrophy in frontal and temporal lobes, with peaks in right middle temporal gyrus ([55, −15, −15] *t*_(106)_ = 9.26, FWE *p* < 0.001), right orbitofrontal gyrus ([38 26 −1] *t*_(106)_ = 8.93, FWE *p* < 0.001), left middle temporal gyrus ([−47, −3, −16] *t*_(106)_ = 8.02, FWE *p* < 0.001), and left frontal operculum ([−38, 22, 0] *t*_(106)_ = 7.76, FWE *p* < 0.001). They showed Bayesian evidence for no atrophy in occipital pole and along superior aspects of the parietal lobe.

As is usual, patients with nfvPPA showed relatively little atrophy, with no voxels surviving FWE *p* < 0.05 correction. With cluster-wise correction at an uncorrected *p* < 0.001 cluster defining height, they showed two clusters of atrophy centered on left and right middle frontal gyrus ([−27 −1 48], peak *t*_(106)_ = 5.55, cluster extent = 14,817, FWE *p* < 0.001; [30 28 37], peak *t*_(106)_ = 5.33, cluster extent = 24,381, FWE *p* < 0.001). They showed widespread bilateral Bayesian evidence for no atrophy in superior temporal gyri, the motor and sensory cortex, superior parietal cortex and occipital lobe.

By contrast, patients with PCA showed marked atrophy in occipital and parietal lobes bilaterally, with peaks in left and right inferior occipital gyri ([−39 −86 2] *t*_(106)_ = 10.81, FWE *p* < 0.001; [43 −83 3] *t*_(106)_ = 7.92, FWE *p* < 0.001). They showed Bayesian evidence for no atrophy in both inferior frontal gyri and in the motor and sensory strips.

Patients with ADMCI showed a typical atrophy with prominent medial temporal loss. They displayed atrophy peaks in left parahippocampal gyrus ([−23 −15 −35] *t*_(106)_ = 8.02, FWE *p* < 0.001) and right hippocampus ([37 −28 −7] *t*_(106)_ = 7.55, FWE *p* < 0.001). They showed Bayesian evidence for no atrophy in superior temporal gyri and inferior frontal gyri.

When all patients were combined and compared against controls, atrophy peaks were observed in right middle temporal gyrus ([54 −17 −17] *t*_(106)_ = 10.52, FWE *p* < 0.001), left supramarginal gyrus ([−47 −48 38] *t*_(106)_ = 9.60, FWE *p* < 0.001), right posterior cingulate ([15 −35 44] *t*_(106)_ = 9.34, FWE *p* < 0.001) and right hippocampus ([36 −30 −4] *t*_(106)_ = 8.40, FWE *p* < 0.001).

### All patient groups show reduced MMN amplitude but preserved responses to standard tones

**Figure 3.**
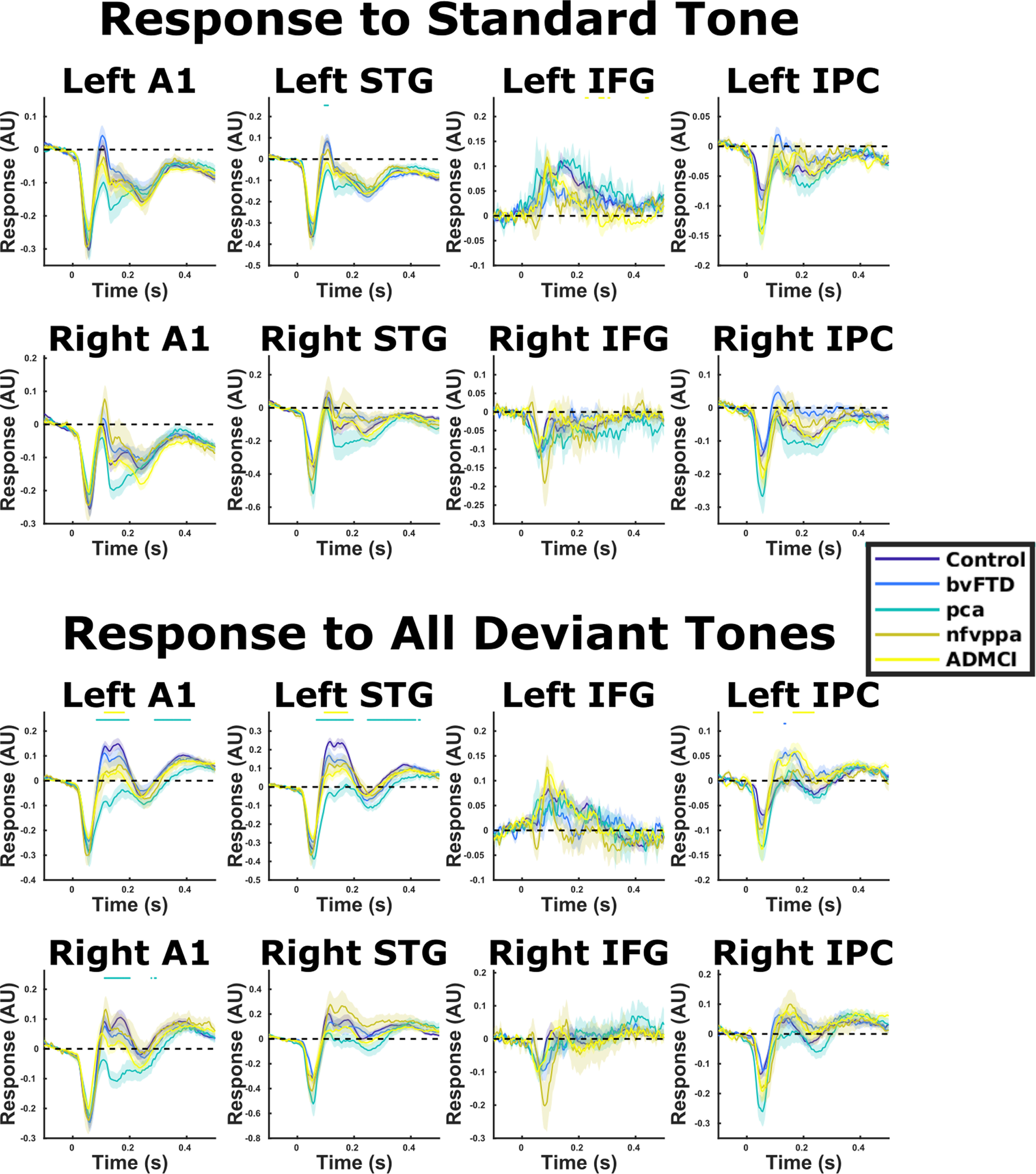
Source-localized evoked responses for standard the average of all standard and all deviant tones. Means and SEs across subjects are plotted, along with an illustrative significance bar, showing time points at which patient group responses significantly differed from control responses, *p* < 0.05 FDR corrected across time points using the Benjamini and Hochberg method. A1, primary auditory cortex; STG, superior temporal gyrus; IFG, inferior frontal gyrus; IPC, inferior parietal cortex. In A1 and STG bilaterally, the response lines for standard (STD) stimuli overly one another for all groups except PCA, which has a slightly more negative trace from 80 to 200 ms. However, in the deviant (DVT) case, the lines separate by group at 100–200 ms, albeit not statistically significantly for nfvPPA and bvFTD groups when FDR corrected across time. Quantifications of this difference are shown in Figure 5.

**Figure 4.**
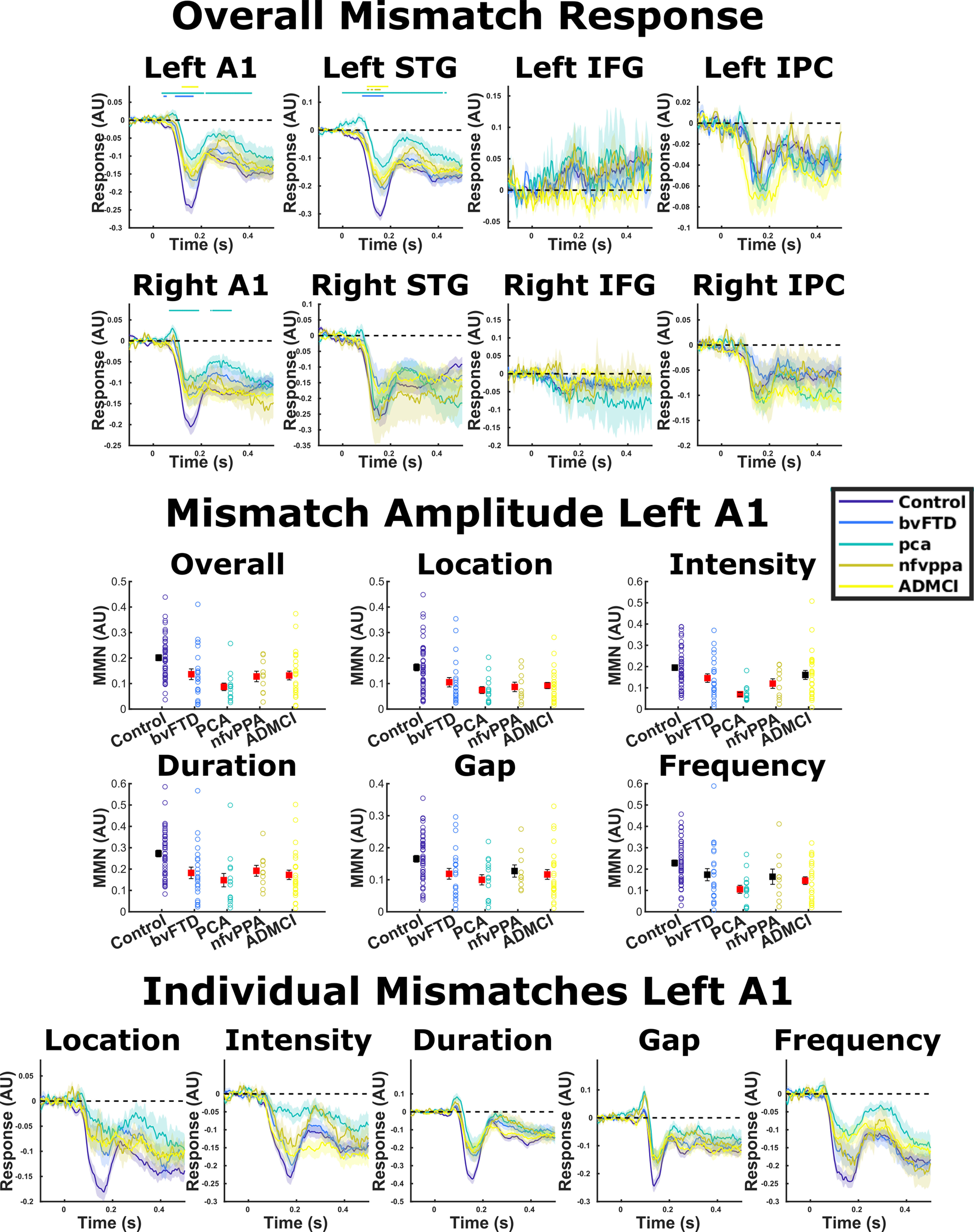
Source-localized evoked responses for MMN. Means and SEs across subjects are plotted, along with an illustrative significance bar, showing time points at which patient group responses significantly differed from control responses, *p* < 0.05 FDR corrected across time points using the Benjamini and Hochberg method. A1, primary auditory cortex; STG, superior temporal gyrus; IFG, inferior frontal gyrus; IPC, inferior parietal cortex. The middle panel shows MMN amplitude averaged between 100 and 200 ms in left A1 (Hughes et al., 2013). One-way ANOVAs in all deviant types showed a main effect of diagnosis. Groups that significantly differed from controls in *post hoc* tests are colored red for each condition. Error bars illustrate standard error of the mean. The bottom panel shows the MMN waveform in left A1 for each deviant type individually.

Source-localized evoked responses for our predefined regions of interest are shown in Figure 3 for each group, for the standard tone and deviant tones combined. A whole-brain SPM contrast confirmed modulation of all of our ROIs by the overall contrast between standard and deviant tones: the strongest mismatch response was around A1 and STG bilaterally (left peak [−42, −18, 20] *F*_(1,590)_ = 82.9, whole-brain FWE *p* < 0.001, right peak [44 −6 16] *F*_(1,590)_ = 53.6, whole-brain FWE *p* < 0.001). There were also significant contrasts with small volume correction (8 mm spheres) at all of our literature-based prespecified co-ordinates of interest: left IFG [−46 20 8] *F*_(1,590)_ = 12.2, FWE *p* = 0.008, right IFG [46 20 8] *F*_(1,590)_ = 19.8, FWE *p* < 0.001, left IPC [−49 −38 38] *F*_(1,590)_ = 13.2, FWE *p* = 0.006, and right IPC [57 −38 42] *F*_(1,590)_ = 12.9, FWE *p* = 0.006. Waveforms for the mismatch waveform at each of these locations are shown in Figure 4, upper panel.

For MMN amplitude in our literature prespecified left A1 ROI (Hughes et al., 2013), repeated measures ANOVA demonstrated a main effect of diagnosis (*F*_(4,118)_ = 6.57, Greenhouse–Geisser *p* = 8.24 × 10e-5; Fig. 4, middle panel). *Post hoc* Tukey–Kramer tests demonstrated that all groups had lower MMN amplitude than controls (*p* = 0.036–*p* = 0.0002). Examining time point by time point, patients with bvFTD had lower MMN amplitude than controls between 44 and 164 ms, patients with PCA between 36 and 208 ms, and patients with ADMCI between 120 and 184 ms. Patients with nfvPPA did not significantly differ at any time point with FDR correction across time, but displayed smaller MMN responses between 48 and 180 ms at an uncorrected *p* < 0.05 threshold. Similar patterns were observed in right A1 and left STG. There was a diagnosis by deviant-type interaction in MMN amplitude (*F*_(16,472)_ = 2.15, Greenhouse–Geisser *p* = 0.0095), but examining the MMN amplitude (Fig. 4, middle panel) and waveform (Fig. 4, lower panel) by condition, there were no striking differences in response profile. Specifically, between-group differences in MMN response amplitude were domain general, being present in all oddball types individually. Additionally, in the whole-brain SPM analysis, contrasting auditory oddball types across all subjects, there were no significant differences in activation, even with small volume correction in our IFG and IPC ROIs.

Our control analysis confirmed there was no group difference in the amplitude of the M100 response to the standard tone (*F*_(4,118)_ = 1.03, Greenhouse–Geisser *p* = 0.395; Fig. 3, upper) or the deviant tone (*F*_(4,118)_ = 1.38, Greenhouse–Geisser *p* = 0.245; Fig. 3, lower), confirming that reductions in MMN amplitude were not simply because of reduced global signal in the patient populations.

**Figure 5.**
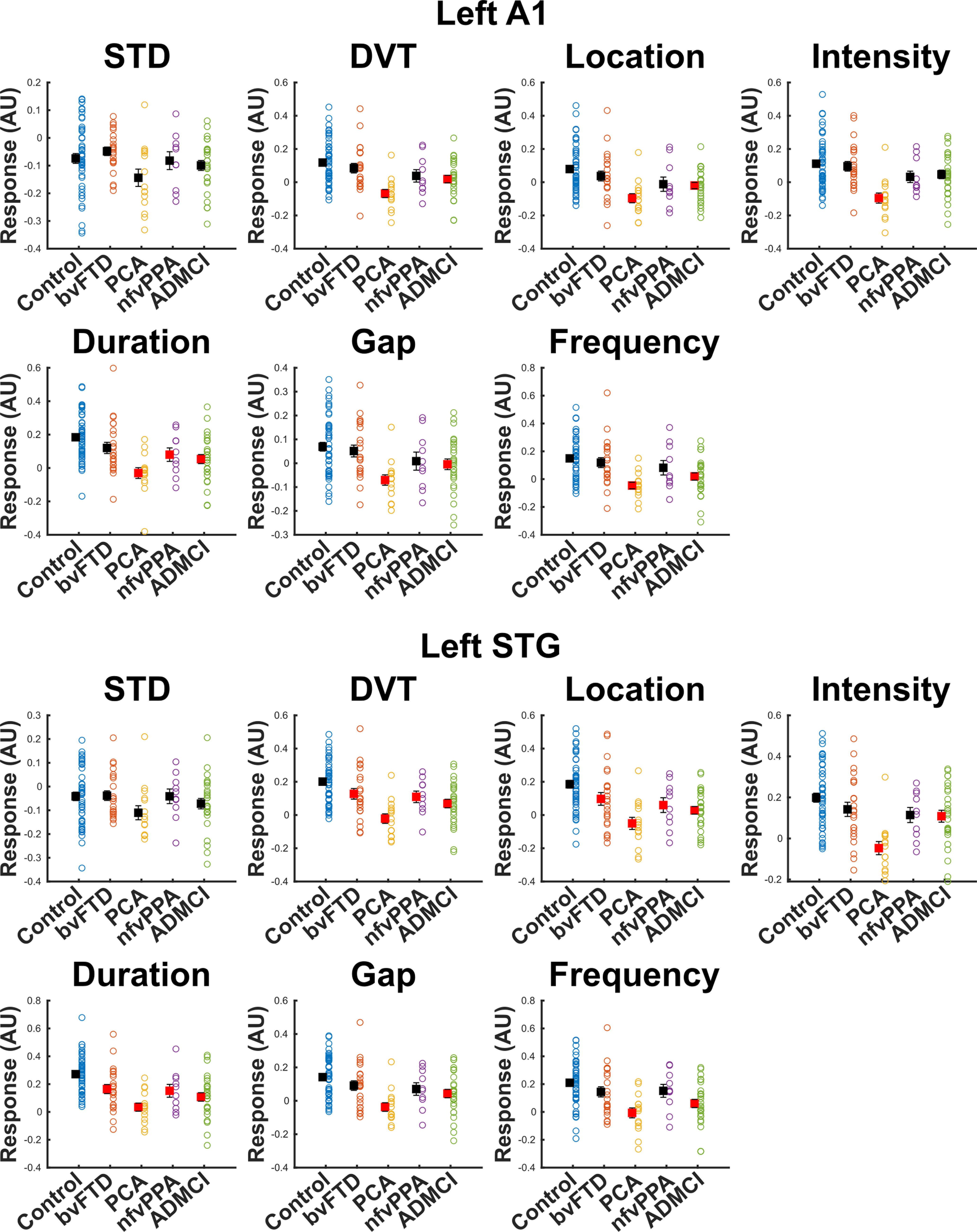
Our literature prespecified approach was to quantify MMN amplitude in left A1 (Hughes et al., 2013) by averaging amplitude from 100 to 200 ms. Individual datapoints are shown, as well as the mean its standard error. To gain insight into whether the differences we demonstrate are primarily driven by adaptation to environmental regularity or response to novel stimuli, we repeated the same procedure with the standard (STD) and combined deviant (DVT) response, as well as for each oddball type individually, and assessed group differences with ANOVA. In left A1, there was no main effect of diagnosis on STD response in the 100- to 200-ms time window (*p* = 0.089). There was a main effect of diagnosis on DVT amplitude (*p* = 1.73 × 10^–5^). *Post hoc* tests demonstrated that patients with PCA and ADMCI had significantly lower amplitudes than controls (illustrated in red), but bvFTD and nfvPPA did not significantly differ from controls. This pattern was broadly the same across deviant types individually, with small changes in pattern resulting in nfvPPA differing from controls in duration deviants and ADMCI not differing in intensity deviants. The data in left STG had slightly better SNR than A1, because of its closer proximity to the scalp. Here, there was still no main effect of diagnosis for STD amplitude (*p* = 0.245). For DVT, there was a main effect of diagnosis (*p* = 6.28 × 10^–7^), and here, we were able to detect a group difference in response amplitude for every group individually versus controls in *post hoc* tests. However, it is important to note that left STG was not our literature specified location of interest for MMN quantification, and is included here only as data illustration, not the basis of any strong claims in the manuscript.

An impaired ability to detect and respond to change might be because of either a failure to adapt to regularities in the environment, reflected in the standard tone, or to an abnormal response to novel stimuli, reflected in the deviant tone, or both. The MMN is a paired difference response, meaning that it is not affected by any nonspecific influences on the modeled ERP within an individual, leaving only the response to change. In our data, between-group differences seem to be primarily driven by the deviant conditions (Fig. 3). Across all time points, there was no significant pairwise difference standard tone response between controls and any other group, but there was a significant difference in deviant tone response between controls and patients with PCA between 84 and 196 and 288 and 412 ms, and patients with ADMCI between 112 and 180 ms. A similar pattern was observed when quantifying overall standard and deviant response amplitudes in the MMN time window (Fig. 5).

Overall, all patient groups had impaired auditory change detection as measured by MMN amplitude in A1, despite very different loci of neurodegeneration. These impairments were domain general, and not driven by a global reduction in signal strength, as might be observed if the changes were simply a marker of atrophy in auditory brain regions.

### Structure-function relationships selectively implicate the MD network

**Figure 6.**
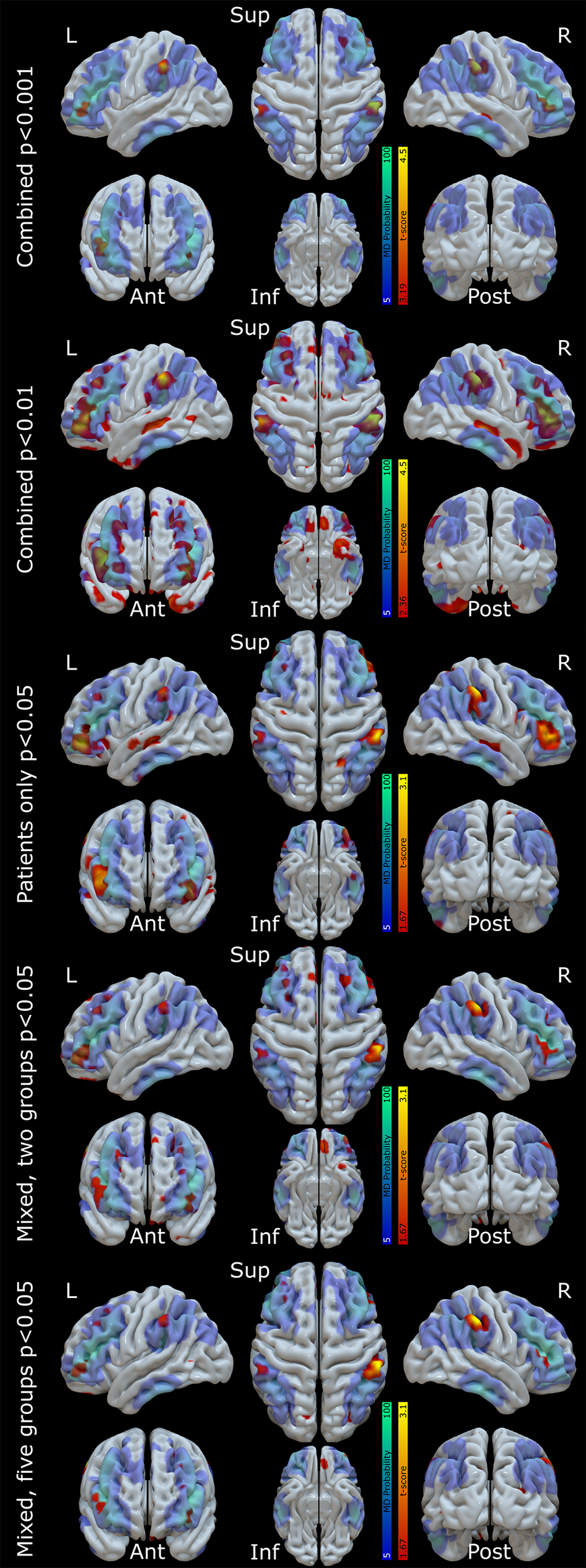
The locations of positive correlation between left A1 MMN amplitude and gray matter volume are shown in “hot” colors for various statistical models. A probabilistic map of the MD (lateral fronto-partietal) network from HCP derived resting state connectivity networks (Dworetsky et al., 2021) is overlaid in “cool” colors. In the first model, all participants are included as a single group, with age and TIV as covariates of no interest, to elucidate the anatomic correlates of auditory MMN amplitude. This model is shown twice, thresholded at *p* < 0.001 for the distributional analyses described in the text, and *p* < 0.01 uncorrected for illustration of more widespread MD involvement below statistical threshold. This regression model's fits in each of our four ROIs (left and right frontal and parietal nodes) are shown in Figure 7, left panel. Next, in the third row, we show the same model with control participants excluded (i.e., patients only), to ensure nonspecific group effects are not the driver of our results. In the fourth and fifth rows, we show linear mixed models, with age and TIV as covariates of no interest, and a factor group with either two (controls, patients) or five (controls, bvFTD, nfvPPA, PCA, ADMCI) levels, to exclude Simpson's paradox. The five-group model's fits in each of our four ROIs are shown in Figure 7, right panel. All models were topographically similar and showed overlapping voxels of peak significance in our ROIs (Table 2).

**Figure 7.**
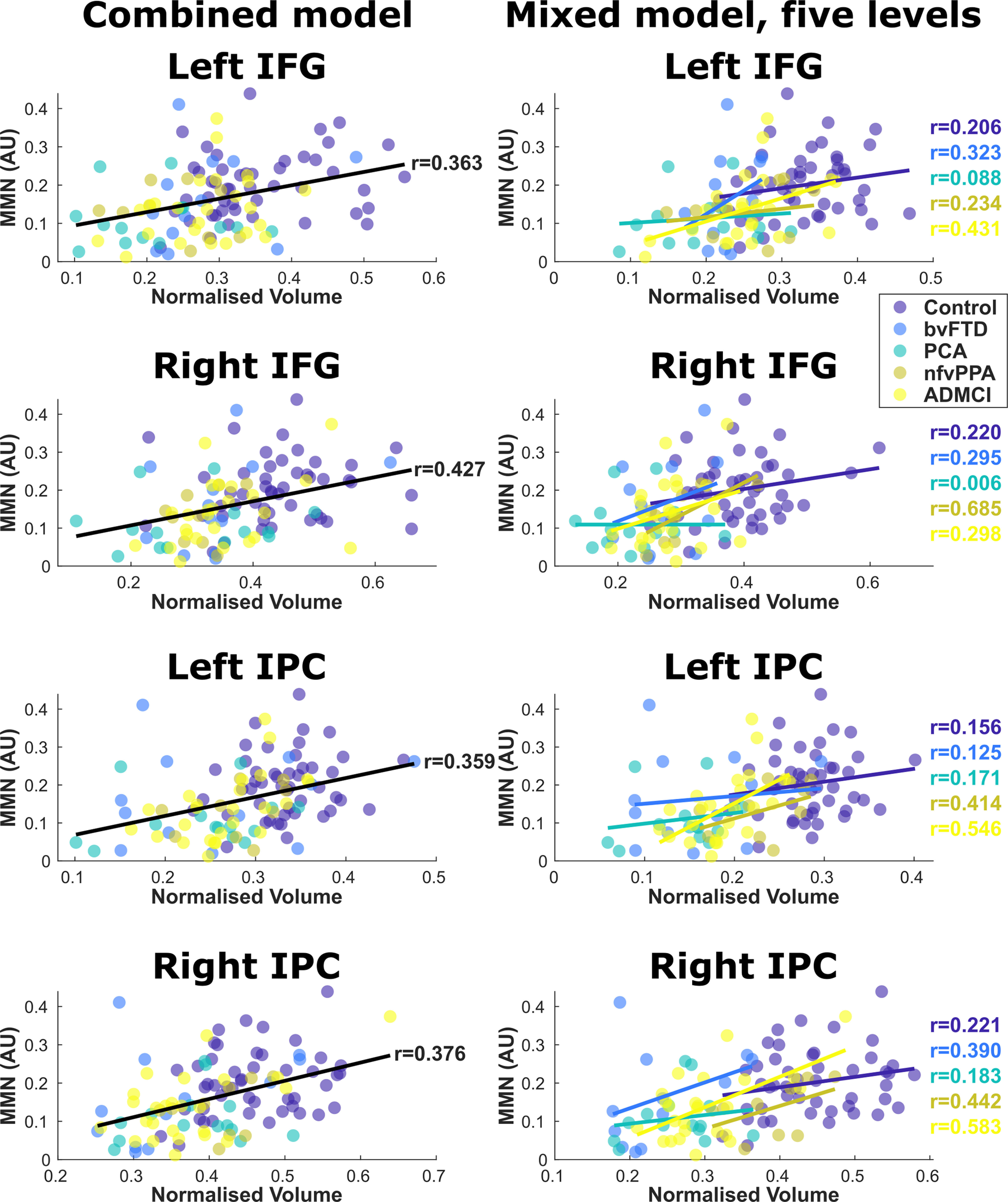
Regression model datapoints in frontal and parietal hot-spot regions, with individuals color-coded by group. IFG, inferior frontal gyrus; IPC, inferior parietal cortex. The left column shows the combined model with all participants included as a single group, with age and TIV as covariates of no interest. The global trend line is shown. The right column shows the linear mixed model with age and TIV as covariates of no interest and a factor group with five levels, demonstrating similar structure-function relationships in every group individually.

To assess the neuroanatomical drivers of reduced MMN amplitude in neurodegeneration we correlated the amplitude of the auditory cortical MMN response against gray matter volume for all participants (Fig. 6, rows 1, 2). Correlations were significant, with reduced MMN amplitude related to atrophy in parietal (left supramarginal *t*_(103)_ = 3.87, FWE *p* = 0.013 at [−61 −35 43], right supramarginal *t*_(103)_ = 4.49, FWE *p* = 0.002 at [60 −30 43]) and frontal (left triangularis *t*_(103)_ = 3.39, FWE *p* = 0.026 at [−36 43 1], right triangularis *t*_(103)_ = 3.55, FWE *p* = 0.016 at [44 39 4]) ROIs (Fig. 7, left).

**Table 2.** Table of structure-function relationships for each regression model individually

Model	Left parietal [−61 −35 43]	Right parietal [60 −30 43]	Left frontal [−36 43 1]	Right frontal [44 39 4]
Combined regression	*t*_(103)_ = 3.87, *p* < 0.001	*t*_(103)_ = 4.49, *p* < 0.001	*t*_(103)_ = 3.37, *p* < 0.001	*t*_(103)_ = 3.55, *p* < 0.001
Combined regression, controls excluded	*t*_(56)_ = 2.20, *p* = 0.016	*t*_(56)_ = 2.85, *p* = 0.003	*t*_(56)_ = 1.43, *p* = 0.078 nearby left frontal peak at [−44 47 −5] *t*_(56)_ = 2.55, *p* = 0.005	*t*_(56)_ = 2.30, *p* = 0.013
Linear mixed model, two groups	*t*_(102)_ = 2.03, *p* = 0.021	*t*_(102)_ = 2.90, *p* = 0.002	*t*_(102)_ = 1.93, *p* = 0.028	*t*_(102)_ = 1.90, *p* = 0.030
Linear mixed model, five groups	*t*_(99)_ = 2.03, *p* = 0.023	*t*_(99)_ = 3.11, *p* = 0.001	*t*_(99)_ = 1.70, *p* = 0.046	*t*_(99)_ = 1.71, *p* = 0.046

Across all three confirmatory models and four ROIs, the voxel of peak significance in the combined regression is significant in eleven of twelve cases. The only exception is the left frontal voxel in the ungrouped regression model with controls excluded, which is only trend significant. However, as shown in the maps (Fig. 6, second row), there is an extensive left frontal cluster in this condition, which is slightly more anterolateral than the original analysis, just missing the original peak. The peak of this cluster is also reported.

Crucially, there were no significant correlations between MMN amplitude and gray matter volume in primary auditory cortices (left *t*_(103)_ = 1.39, FWE *p* = 1, right *t*_(103)_ = 0.89, FWE *p* = 1) or superior temporal gyri (left *t*_(103)_ = 0.32, FWE *p* = 1, right *t*_(103)_ = 1.38, FWE *p* = 1), confirming that impaired auditory change detection was not simply because of neurodegeneration in auditory brain regions. Nor were there any significant correlations at any of the overall atrophy peaks across all participants that fell outside of MD areas (middle temporal gyrus, posterior cingulate, and hippocampus), confirming that the structure-function relationship we report here is not simply picking out the most commonly atrophic cortical regions in our patients. This lack of nonspecific group effects was confirmed by additional analysis excluding the control participants (Table 2; Fig. 6, row 3), and was shown to be consistent across all groups individually (Table 2; Fig. 6, rows 4, 5 Fig. 7, right).

We tested the specificity of these analyses by comparing the overlap of the structure-function map from our overall regression model with independent data. The peak correlations of all of clusters fell within Idan Blank's publicly available MD functional parcellation (Shain et al., 2020), based on 197 participants performing a spatial working memory task. However, binary parcellations run the risk of confusing functional networks that are spatially overlapping at the group level but segregated at the single subject level (Fedorenko and Blank, 2020). To address this concern, we compared voxelwise overlap of our whole-brain maps thresholded at *p* < 0.001 against probabilistic maps of the MD and superficially similar language networks derived from functional localizers, thresholded at 5% between-subject agreement (Woolgar et al., 2018). Our data showed a 67.9% overlap with the MD contrast and only a 9.2% overlap with the language contrast. Additionally, we repeated this analysis against published resting state networks derived from the human connectome project (Dworetsky et al., 2021; Fig. 6). Our structure-function maps showed a 72.9% overlap with their MD (fronto-parietal) network, much more than with the superficially similar cingulo-opercular (26.4%), salience (39%), and language (22.6%) networks.

Thresholding in this way may obscure differences in the strength of structure-function relationships and the probability of individual activations with the functional localizer or resting state connectivity network membership. We therefore calculated an activation ratio by combining the maps multiplicatively, normalized against size and range. Against the functional localizers, our structure-function maps showed a normalized activation ratio of 0.54 for the MD contrast, and only 0.17 for the language contrast. Against the resting state networks, our structure-function maps showed a normalized activation ratio of 0.70 for the MD network, and only 0.19 for the cingulo-opercular network, 0.01 for the salience network, and 0.26 for the language network.

Finally, as a control analysis to ensure that these results were not a nonspecific effect of there being more atrophy in MD than language areas at the group level, we re-calculated these ratios from structural maps alone. Reassuringly, against the functional localizer this resulted in very similar normalized ratios of 0.19 for the MD network and 0.24 for the language network, and against the resting state networks this resulted in a normalized ratio of 0.18 for the MD network, 0.19 for the cingulo-opercular network, 0.17 for the salience network, and 0.22 for the language network. Additionally, we show in (Fig. 6, row 2) that the specificity of our finding is not the product of an arbitrary statistical thresholding. Reducing the threshold for structure-function relationships resulted in more widespread peaks that filled a larger proportion of the MD map and continued to spare both auditory areas and heavily atrophic areas (middle temporal gyrus, posterior cingulate and hippocampus) outside of MD.

Overall, therefore, the loci best explaining impaired change detection are outside of auditory cortex, strongly overlap with the MD system, and are not simply those areas that are most atrophic overall.

### Frontal and parietal loci both show effective connectivity with auditory regions during MMN

To understand the functional role of frontal (IFG) and parietal (IPC) cortex in generating the MMN response observed in temporal cortex, we assessed effective connectivity with DCM (Fig. 1). Across all subjects, the exceedance probability (xp) of the fully connected model 32 was 0.92. No other models had an xp above 0.05. This model was also the single most likely generative model for controls alone (xp = 0.80, with the second placed model omitting direct connectivity between IPC and IFG, xp = 0.16), in ADMCI (xp = 0.52, with the second placed model lacking only cross-hemispheric connections xp = 0.14), and in PCA (xp = 0.54, with the second placed model being fully connected again except for direct connectivity between IPC and IFG, xp = 0.17). In bvFTD the single most likely model was fully connected except for absent inter-hemispheric connections (xp = 0.45), with the fully connected model a close second (xp = 0.39). In nfvPPA the models with the highest probabilities were fully connected except for absence of direct connectivity between IPC and IFG (xp = 0.33), cross-hemispheric connections (xp = 0.12) or cross-hemispheric and STG <-> IPC connections (xp = 0.13). Overall, therefore, every model with an xp of >0.05 included both IFG and IPC bilaterally.

We confirmed this finding with a model family comparison using Bayesian model averaging (Penny et al., 2010) across eight model families, each including four models with the same connectivity between IFG, IPC, and STG. Across all subjects (xp = 0.98), and within the control (xp = 0.84), bvFTD (xp = 0.94), and ADMCI (xp = 0.82) groups individually, there was strong evidence for the family of models including full bilateral connectivity between IPC, IFG, and STG. This model family was also likely in PCA (xp = 0.57) and nfvPPA (xp = 0.43), but in these conditions, there was additional evidence (PCA xp = 0.36, nfvPPA xp = 0.43) for the model family in which IPC and IFG were not connected to each other (but were still connected to STG). Overall, there was no evidence for widespread disconnection of fronto-parietal regions from STG in neurodegeneration, demonstrating their continued involvement in the MMN response even in the face of atrophy.

### Atrophic frontal and parietal loci have reduced top-down influence, but preserved nodes increase in effective connectivity

**Figure 8.**
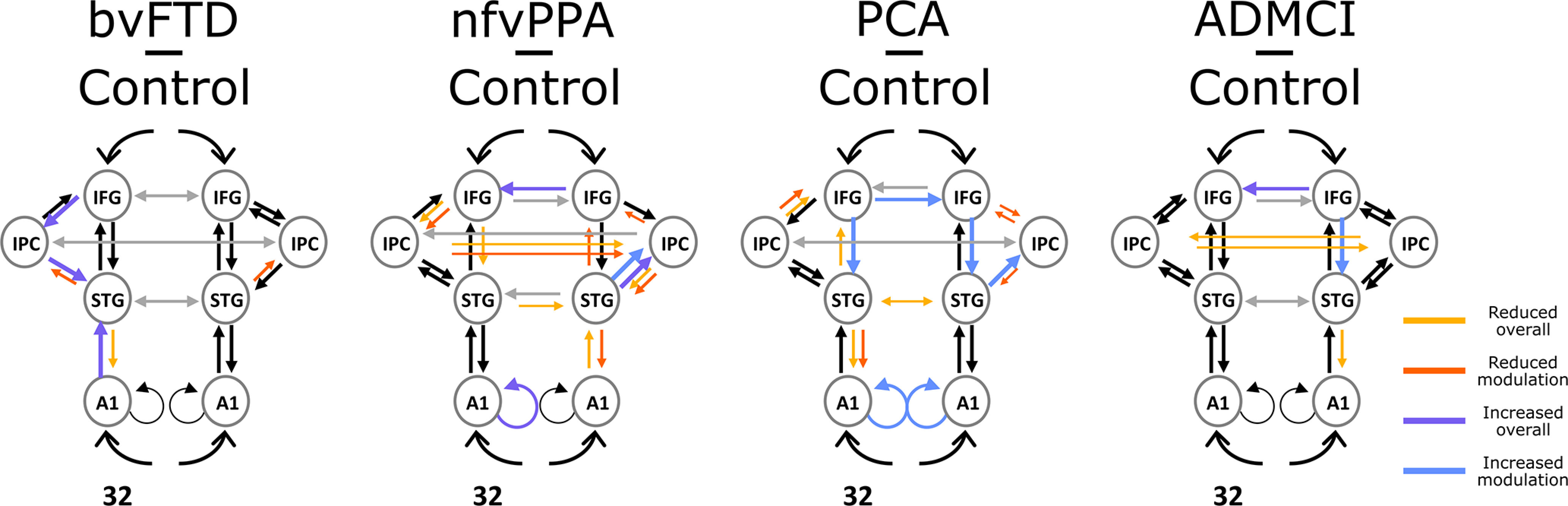
Differences between patients and controls in effective extrinsic connectivity strength and auditory-change related modulation with Bayesian probabilities >0.95. “Modulation” differences represent changes in effective connectivity as a function of the standard (STD)-deviant (DVT) contrast (i.e., the group by condition interaction), while “overall” differences represent changes in effective connectivity across all trial types. Larger colored arrows represent strengthening in patients compared with controls, while smaller colored arrows represent weakening. A1, primary auditory cortex; STG, superior temporal gyrus; IFG, inferior frontal gyrus; IPC, inferior parietal cortex.

To assess group differences in the strength of effective extrinsic connectivity between patients and controls, both in terms of connection strength and auditory-change related modulation, we compared the strength of connections in the winning (fully connected) model with PEB. Patients differed from controls in the strength of forward and backward effective connectivity, and their modulation by auditory change (Fig. 8). All groups showed a reduction of backward connectivity from STG to A1 connectivity in at least one hemisphere.

Changes in connectivity among association cortex differed according to disease group and the most widespread connectivity abnormalities were in the more focal neurodegenerative syndromes that selectively involved frontal or parietal regions. PCA, with predominant occipital-parietal neurodegeneration, showed reduced task-modulation of connectivity bilaterally from IPC to IFG, and to right STG; and in contrast, significantly increased top-down modulatory influence of IFG on STG bilaterally. There was reduced left STG to A1 modulation, and bilaterally increased local (self) modulation of A1. nfvPPA, with left IFG/Insular neurodegeneration, showed reduced modulation of connections from left IFG to IPC, reduced left IFG to STG connectivity, and a widespread increase in right-sided and reduction in left-sided influence.

Overall, this analysis shows that neurodegeneration reduced top-down effective connectivity from affected MD nodes and, in the more focal syndromes of nfvPPA and PCA, increased effective connectivity from unaffected MD nodes. This compensation was only partially effective, as overall top-down influence on A1 fell in all groups.

### Neurodegeneration increases functional connectivity in a frequency-specific manner

**Figure 9.**
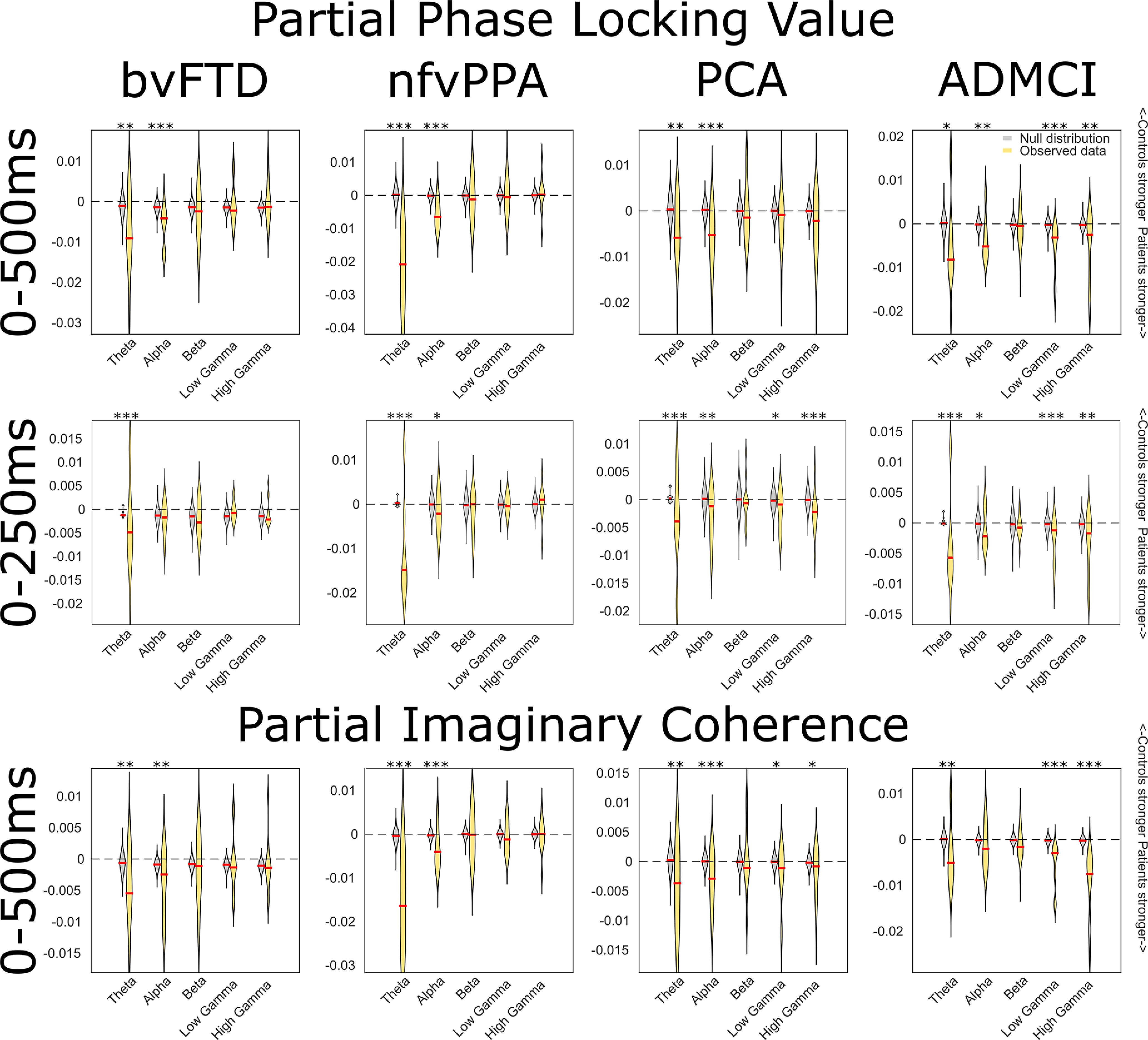
Frequency resolved connectivity analyses with phase-locking value (plv) and imaginary coherence (icoh). For each frequency band and for each connection, the observed mean plv or icoh in the relevant patient group was subtracted from the same measure in the control group. Negative values indicate stronger plv or icoh in patients than controls. These data were compared with a null distribution of 1100 values for each frequency band (100 iterations at each of 11 unique connections), generated from the same subtraction for trial-shuffled connectivity, using two-sample Welch's *t* tests. *p < 0.05, **p < 0.01, ***p < 0.001. The pattern of results was similar for continuous data epoched 0–500 ms after tone onset, and discontinuous data focused on the time of the MMN, 0–250 ms after tone onset. These findings cannot be accounted for by overall differences in power between groups; in general, patients had the same or higher functional connectivity, and the same or lower power compared with controls (Fig. 12).

Next, we assessed the frequency specificity of the connectivity changes in the networks, using partial phase-locking value and partial imaginary coherence. First, we quantified the strength of each connection in each patient group, relative to controls, and compared this to a permutation-based null distribution (Fig. 9). Concordant with Sami et al. (2018), we found that connectivity changes in bvFTD and nfvPPA were restricted to lower frequencies, while in ADMCI and PCA additional connectivity changes were found in higher frequencies. No group showed an overall difference across all nodes in the β band, but many individual connections lay outside of the null distribution. Significant differences were all in the direction of stronger average phase-locking value and imaginary in patients than controls.

**Figure 10.**
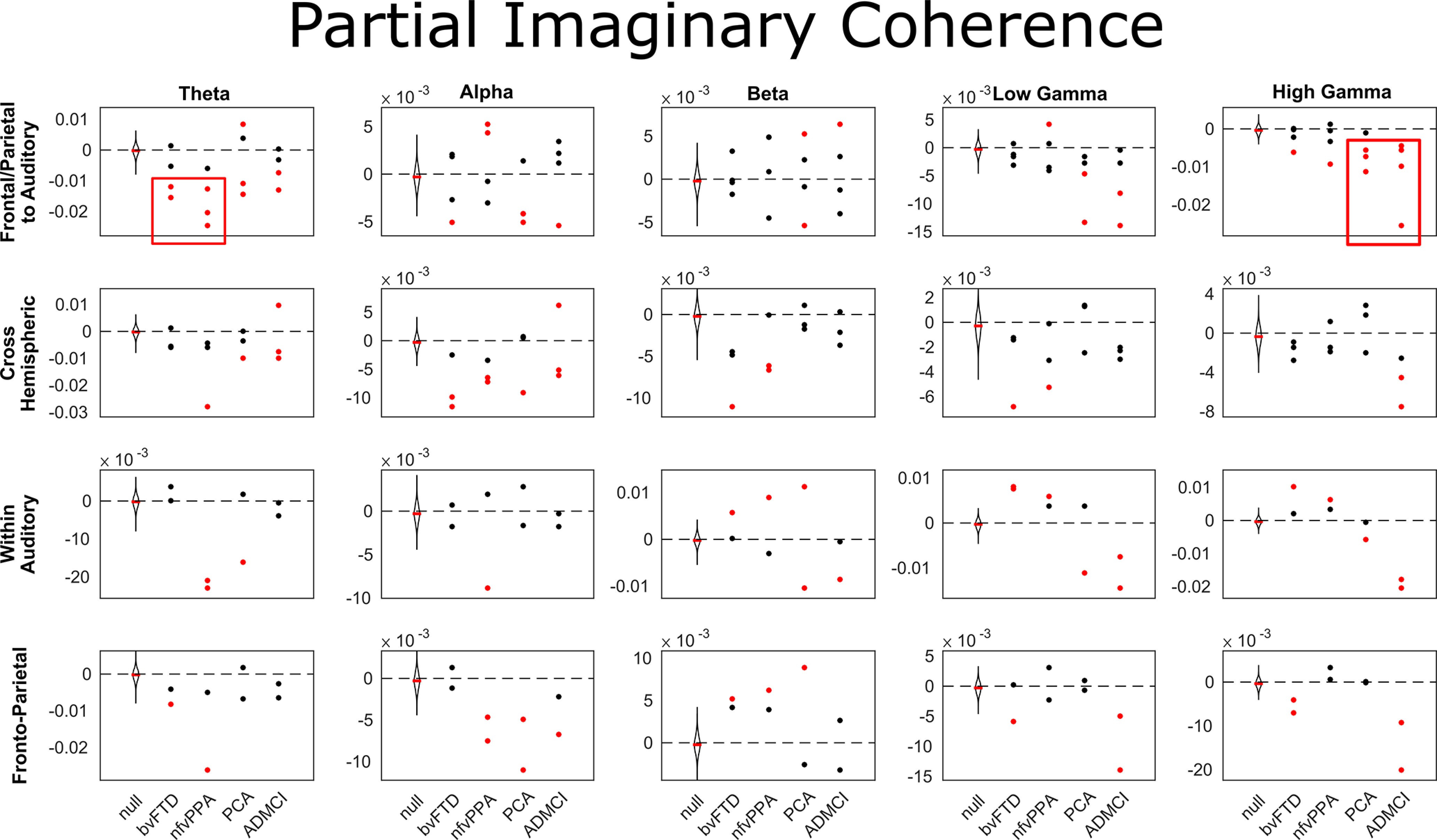
Partial phase locking value for individual connections, broken down by group and network. Negative values indicate stronger partial phase locking value in patients than controls, and vice versa. Red boxes highlight consistently increased functional connectivity between MD and auditory regions in low frequencies for patients with bvFTD and nfvPPA, and high frequencies for PCA and ADMCI. MD to auditory connections were STG-IPC and STG-IFG on each side, cross hemispheric connections were STG-STG, IPC-IPC, and IFG-IFG, within auditory connections were A1-STG in each hemisphere, and MD region connections were IPG-IFG in each hemisphere. A1, primary auditory cortex; STG, superior temporal gyrus; IFG, inferior frontal gyrus; IPC, inferior parietal cortex. These data were compared with a null distribution of 400 iterations with trial-shuffled connectivity per connection (100 iterations for each of the four groups). Individual connections were colored red if they lay completely outside of this null distribution (equivalent to a permutation *p*-value of <0.01 after Bonferroni correction across frequency bands).

**Figure 11.**
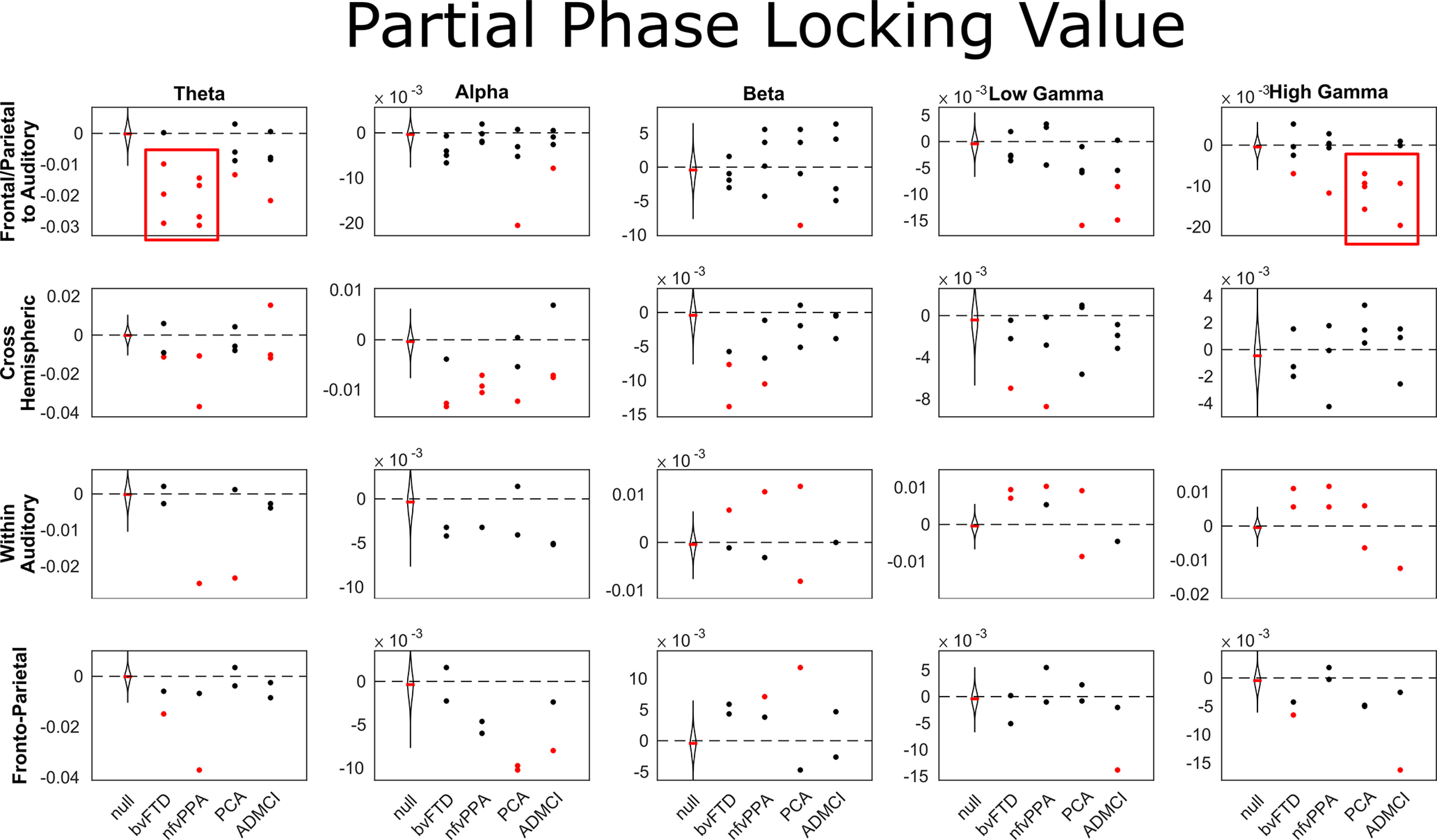
Partial imaginary coherence for individual connections, broken down by group and network. Negative values indicate stronger imaginary coherence in patients than controls, and vice versa. MD to auditory connections were STG-IPC and STG-IFG on each side, cross hemispheric connections were STG-STG, IPC-IPC, and IFG-IFG, within auditory connections were A1-STG in each hemisphere, and MD region connections were IPG-IFG in each hemisphere. A1, primary auditory cortex; STG, superior temporal gyrus; IFG, inferior frontal gyrus; IPC, inferior parietal cortex. These data were compared with a null distribution of 400 iterations with trial-shuffled connectivity per connection (100 iterations for each of the four groups). Individual connections were colored red if they lay completely outside of this null distribution (equivalent to a permutation *p*-value of <0.01 after Bonferroni correction across frequency bands).

Next, we compared frequency-resolved connectivity between auditory nodes, fronto-parietal (MD) nodes, connections between auditory and MD nodes, or across hemispheres. Results were very similar for phase-locking value (Fig. 10) and imaginary coherence (Fig. 11). Connectivity abnormalities between auditory and MD regions were marked in the low-frequency θ band in bvFTD and nfvPPA, and in the high-γ band in PCA and ADMCI.

**Figure 12.**
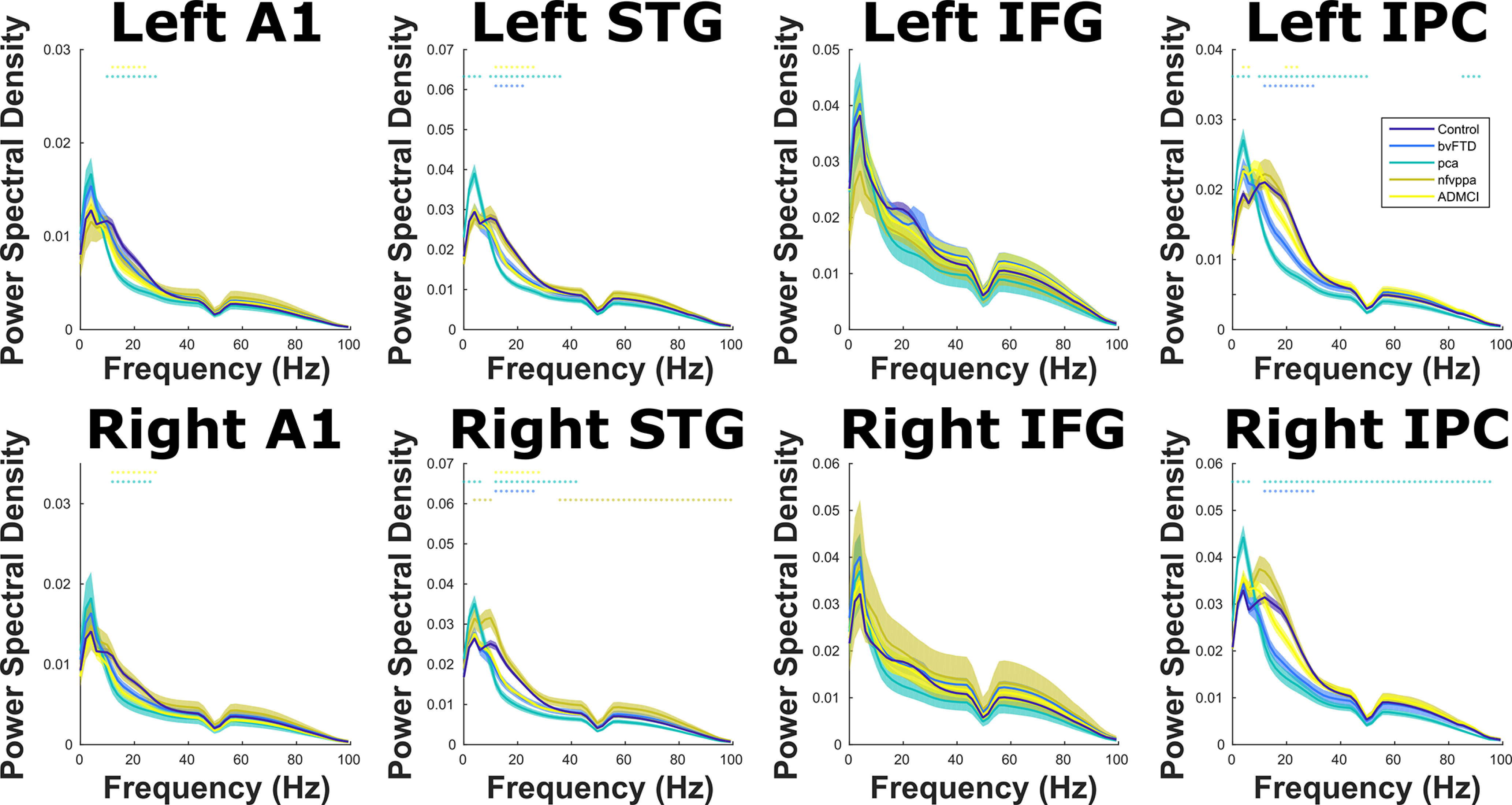
Power spectral density over the whole experiment in each ROI, color coded by group. Means and SEs across subjects are plotted, along with illustrative significance dots, showing frequency steps at which patient group power significantly differed from controls, *p* < 0.05 FDR corrected across frequency using the Benjamini and Hochberg method. Note that the data have been filtered to exclude 50-Hz line noise, and the multitaper decomposition uses a ±4-Hz smoothing box.

These results were not driven by overall differences in power and resultant changes in signal-to-noise ratio (Aru et al., 2015); in general, patients had the same or lower power compared with controls (Fig. 12). The exceptions were that patients with PCA had higher θ power than controls in STG and IPC bilaterally, and patients with nfvPPA had higher power than controls in right STG across α and γ bands. These were not the frequency bands in which most of the increased auditory-MD functional connectivity was observed; patients with PCA had most significant increases in the γ band, while those with nfvPPA had most significant increases in the θ band.

## Discussion

Here, we provide convergent evidence for a causal role in auditory change detection for bilateral, ventral frontal and parietal loci within the MD network, by combining structure-function mapping, DCM, and frequency-resolved functional connectivity analyses across a range of neurodegenerative diseases. Key outcomes were (1) confirmation that multiple foci of neurodegeneration impaired auditory change detection (Figs. 2, 4); (2) the impairments were domain general, over deviation of frequency, location, loudness, duration, or timbre (Fig. 4); (3) crucially, the loci best explaining impaired auditory change detection were outside of auditory cortex, overlapped selectively with the MD system, and were not simply those areas that are most atrophic (Fig. 6); (4) whereas there is no evidence for widespread disconnection of fronto-parietal regions, there is widespread abnormality of effective connectivity within the MD network, and between it and auditory temporal regions (Fig. 8); (5) across all connections, there is stronger average phase locking and imaginary coherence in patients than controls, consistent with attempted compensation (Fig. 9); (6) connectivity abnormalities manifest in low-frequency bands in patients with Fronto-temporal lobar degeneration (FTLD) pathology, and both low- and high-frequency bands in patients with AD pathology (Fig. 9). These changes were most prominent in those connections between auditory (STG) and MD (IFG and IPC) regions (Figs. 10, 11).

### Environmental change detection requires top-down control from the MD network

The detection of sensory regularities is a fundamental neurocognitive process across the lifespan. However, the ability to detect a change or deviation from such regularities changes with age (De Kerangal et al., 2021), and in many neurologic and psychiatric disorders (Kocagoncu et al., 2020). This impaired ability to recognize and adapt to unexpected events and environmental changes is proposed to be a core feature of dementia (Goto et al., 2014; Lee et al., 2016), but its behavioral assessment is complicated by more general dementia-related impairments, such as problems with working memory and concentration. MMN paradigms (Näätänen et al., 2007) operate independently of attention and are sensitive to the early effects of neurodegenerative diseases (Pekkonen, 2000; Brønnick et al., 2010; Näätänen et al., 2011; Mowszowski et al., 2012; Jiang et al., 2017). They can reveal network-level changes in brain connectivity between temporal auditory regions and domain-general loci in frontal lobes (Hughes et al., 2013; Hughes and Rowe, 2013; Phillips et al., 2015, 2016). These effects are often modeled with a single top-down influence from right frontal cortex, but here we show that this perceptual control (Ptak, 2012) is provided by more widespread dorsal fronto-parietal networks (Kim, 2014), although the “task” is simple and passive.

### Frontal and parietal nodes are interconnected, and both are required for normal mismatch response

The parietal cortex is modulated by unexpected stimuli (Molholm et al., 2005; Dietz et al., 2014; Kompus et al., 2020), and changes in auditory temporal structure (Andreou et al., 2015). Previous casual evidence for parietal damage leading to an impaired ability to respond to environmental change has largely been provided by the poststroke neglect and extinction syndromes, which usually results from lesions to nondominant and dominant IPC, respectively. The specificity of this structure-function relationship was difficult to assess, because the complete unilateral destruction of a network node and its underlying white matter results in widespread imbalance in interhemispheric and fronto-parietal connectivity (Corbetta and Shulman, 2011). Similar findings have been reported from electrophysiological assessments of patients with prefrontal stroke (Deouell and Knight, 2005). Our data provide a much more nuanced and sensitive assessment in a large cohort of individuals who do not have “lesions,” but rather display differing degrees of relative neurodegeneration across implicated network nodes. This allows us to visualize abnormal connectivity between these nodes and the rest of the network, without the forced widespread reorganization induced by their absence. Reduced MMN amplitude in all patient groups, despite selective and differential nodal atrophy or sparing, confirms that degeneration of any of the ventral frontal and parietal nodes of the MD network is sufficient to impair change detection in auditory cortex, while degeneration outside of these nodes does not do so. We do not give primacy to prefrontal or parietal nodes, but rather emphasize their interconnected nature, and show that neurodegeneration anywhere in the MD network results in impaired auditory cortical response to change, but neurodegeneration elsewhere does not.

### Neurodegeneration increases functional connectivity in the task state, but this is not sufficient to rescue effective connectivity from affected nodes

Our frequency-resolved analyses are concordant with both resting state studies in similar patients (Poza et al., 2008; Sami et al., 2018) and preclinical studies in animal models (Kurudenkandy et al., 2014; Koss et al., 2016; Stoiljkovic et al., 2016), providing criterion validity not only for our own work but also for those simpler models of disease, supporting their use in interventional studies. Our analyses and theirs all show Alzheimer-type pathology preferentially affecting high-frequency bands and FTLD pathology affecting only low-frequency bands. Resting state analyses universally show reductions in functional connectivity. However, this pattern is often reversed when network-level connectivity is driven by stimulus or task; as here, patients often display increased frequency-resolved coherence between sources in the same bands (Hughes and Rowe, 2013; Cope et al., 2017), but still show reduced or delayed causal influence. This is likely to represent a compensatory upregulation as neuronal hubs increase their firing rate in the face of neurodegeneration (Maestú et al., 2015), but this mitigation is only partial (Geranmayeh et al., 2014), and breaks down as disease progresses (Jones et al., 2016; Cope et al., 2018), perhaps because the top-down influences arrive too slowly, or neurodegeneration results in a loss of signal precision and accuracy. Here, we use complementary model-based DCM of the evoked response, and model-free frequency-resolved analyses of the induced response to demonstrate this phenomenon in action: coherence increases are most prominent in the connections between auditory and MD nodes, and yet these same connections have reduced effective connectivity.

### Limitations

The primary limitations of this study are not methodological. We have a far larger group size than most functional imaging studies in dementia outside of the resting state, including a diverse range of pathologies and neurodegenerative profiles covering the whole brain, with biomarker evidence of Alzheimer pathology in our MCI group. We employ three radically different and independent techniques (structure-function mapping, DCM, and frequency-resolved functional connectivity analyses) with convergent results, providing reassurance that our findings are not an artifact of a methodological idiosyncrasy. Studies like ours have been criticized for reverse inference when drawing an association between anatomic localizations and previous functional imaging findings in healthy individuals, without localizing individual MD maps (Blank et al., 2017). Domain-general regions, like those examined here in frontal and parietal cortex (Fedorenko et al., 2013), by their nature subserve a wide variety of cognitive tasks. At the individual level, complex maps are revealed of neuronal subpopulations specialized for particular tasks, intermingled with those with a more domain-general role (Fedorenko et al., 2012; Fedorenko and Blank, 2020). Given the degree of smoothing inherent in the atrophy patterns of neurodegenerative disease and methodologically in MEG source reconstructions, how specific can we be about our core conclusion that the MD network is the core driver of our findings? A significant advantage we have here over stroke lesion studies is that our patients have graded and partial pathology that, across the whole cohort, covers widespread brain networks, and implicates multiple loci either simultaneously or differentially. This allowed us to show strong and specific structure-function relationships that overlapped specifically with MD regions and not language, cingulo-opercular or salience networks, despite these networks overlapping in some loci, and all being equally atrophic across the overall cohort. We are able to go further than previous functional connectivity studies in schizophrenia, which have demonstrated a more general cognitive role for MD and cingulo-opercular networks (Sheffield et al., 2015), because of the differential neurodegeneration between individuals in our cohort, clustering by diagnosis, with easily observable group-level differences in our parametric outcome measure (the MMN response). Our data do, however, contain a degree of smoothness, and therefore cannot speak to any potential dissociations between the functions of frontal and parietal subregions in change detection, attention and task switching. In addition, our use of continuous data epoched into a 0- to 500-ms time window for some functional connectivity analyses means that these results may include contributions from re-orienting processes (Schröger, 2005; Kushnerenko et al., 2013). We therefore use the term MD network inclusively, but our findings could equally be interpreted as causal evidence for the ventral attention network if one were to view this as distinct from both the lateral fronto-parietal MD and cingulo-opercular networks (Corbetta et al., 2008). Our own view would be to support a simplification of the taxonomy of fronto-parietal brain networks; here we have confirmed that the same MD network nodes are implicated in perceptual control (Dosenbach et al., 2008; Vincent et al., 2008), an executive function (Seeley et al., 2007; Niendam et al., 2012), and these roles likely combine to support fluid intelligence and working memory (Assem et al., 2020b).

In conclusion, overall, we show that MD regions in bilateral ventral frontal and parietal cortex causally mediate auditory change detection across a range of domains, demonstrating that the MD network plays a role in the “simple task” of automatic sensory change detection through the impairment of top-down control mechanisms. Dementia-related reductions in the neurophysiological amplitude of the auditory mismatch response are not a simple reflection of global atrophy, but are rather a specific manifestation of neurodegeneration in these frontal and parietal regions. Neurodegeneration reduced top-down effective connectivity from affected fronto-parietal nodes and increased effective connectivity from unaffected fronto-parietal nodes. This compensation was only partially effective, as overall top-down influence on STG fell with the involvement of any frontal or parietal node set. All degenerative nodes showed increased functional connectivity, again consistent with compensatory upregulation (Maestú et al., 2015). This increase was frequency-specific, and dissociated by underlying neuropathology concordantly with previous resting state analyses (Sami et al., 2018).
